# Diagnostic accuracy of coronary computed tomography angiography-derived fractional flow reserve

**DOI:** 10.1186/s12938-021-00914-3

**Published:** 2021-08-04

**Authors:** Wenbing Jiang, Yibin Pan, Yumeng Hu, Xiaochang Leng, Jun Jiang, Li Feng, Yongqing Xia, Yong Sun, Jian’an Wang, Jianping Xiang, Changling Li

**Affiliations:** 1 Department of Cardiology, The Third Clinical Institute Affiliated to Wenzhou Medical University, Wenzhou, China; 2 ArteryFlow Technology Co., Ltd, Hangzhou, China; 3 Department of Cardiology, The Second Affiliated Hospital of Zhejiang University School of Medicine, 88 Jiefang Road, Hangzhou, 310009 China; 4 Department of Cardiology, Affiliated Jinhua Hospital, Zhejiang University School of Medicine, Jinhua, China

**Keywords:** Coronary computed tomography angiography, Fractional flow reserve, Computational fluid dynamics, CT-derived FFR

## Abstract

**Background:**

Fractional flow reserve **(**FFR) is a widely used gold standard to evaluate ischemia-causing lesions. A new method of non-invasive approach, termed as AccuFFRct, for calculating FFR based on coronary computed tomography angiography (CCTA) and computational fluid dynamics (CFD) has been proposed. However, its diagnostic accuracy has not been validated.

**Objectives:**

This study sought to present a novel approach for non-invasive computation of FFR and evaluate its diagnostic performance in patients with coronary stenosis.

**Methods:**

A total of 54 consecutive patients with 78 vessels from a single center who underwent CCTA and invasive FFR measurement were retrospectively analyzed. The CT-derived FFR values were computed using a novel CFD-based model (AccuFFRct, ArteryFlow Technology Co., Ltd., Hangzhou, China). Diagnostic performance of AccuFFRct and CCTA in detecting hemodynamically significant coronary artery disease (CAD) was evaluated using the invasive FFR as a reference standard.

**Results:**

Diagnostic accuracy, sensitivity, specificity, positive predictive value (PPV) and negative predictive value (NPV) for AccuFFRct in detecting FFR ≤ 0.8 on per-patient basis were 90.7, 89.5, 91.4, 85.0 and 94.1%, respectively, while those of CCTA were 38.9, 100.0, 5.71, 36.5 and 100.0%, respectively. The correlation between AccuFFRct and FFR was good (*r* = 0.76 and *r* = 0.65 on per-patient and per-vessel basis, respectively, both *p* < 0.0001). Area under the curve (AUC) values of AccuFFRct for identifying ischemia per-patient and per-vessel basis were 0.945 and 0.925, respectively. There was much higher accuracy, specificity and AUC for AccuFFRct compared with CCTA.

**Conclusions:**

AccuFFRct computed from CCTA images alone demonstrated high diagnostic performance for detecting lesion-specific ischemia, it showed superior diagnostic power than CCTA and eliminated the risk of invasive tests, which could be an accurate and time-efficient computational tool for diagnosing ischemia and assisting clinical decision-making.

## Introduction

Accurate diagnosis of stenosis severity is essential for doctors in therapeutic decision-making regarding the need for percutaneous coronary intervention (PCI) or coronary artery bypass grafting (CABG). Coronary computed tomography angiography (CCTA) has emerged as a useful tool for evaluating coronary artery disease (CAD) severity [1]. However, the anatomic information obtained by CCTA is unable to ultimately reflect the physiological and physical influence on blood flow, which results in the poor correlation between lesion-specific ischemia and stenoses detected by CCTA [2–4]. Invasive fractional flow reserve (FFR), which assesses the ratio of flow across stenoses to putative flow in the absence of stenosis, is a well-established reference standard for evaluating the ischemic potential of individual lesions [5, 6]. Several validation studies have demonstrated that FFR-guided coronary intervention can enhance survival and reduce unnecessary revascularization [3, 7]. Despite the clinical benefits of FFR, the cost and potential complication of a pressure wire limited the wide application of FFR. Thus a tool that could precisely calculate the FFR value inexpensively would benefit more people in the world.

Computational fluid dynamics (CFD), as applied to CCTA images, represents a novel technique for assessing the physiological significance of CAD [8]. CT-derived FFR can be computed non-invasively from standard CCTA images without modifying acquisition protocols or additional imaging, and it has been validated in several trials with good diagnostic performance [9–11]. CT-FFR involves coronary segmentation, estimation of blood flow, numerical solving for the flow field across a coronary tree, etc. The complexity of these steps results in the most significant restriction of CT-FFR, namely time-consuming. In the FFR_CT_ computation process, parallel supercomputers are needed [12]. Thus, a CT-derived FFR approach without high computational demands becomes very attractive.

In this study, a novel CT-derived FFR method, AccuFFRct, which efficiently computes lesion-specific non-invasive FFR based on anatomic and functional information, was used to assess its feasibility and diagnostic accuracy with wire-based FFR as a reference standard.

## Results

### Patient characteristics

The study population consisted of 54 patients with 78 vessels, including 34 males and 21 females. 45 (58%) lesions were located in left main or left anterior descending arteries, 10 (13%) were in left circumflex arteries and 14 (18%) were in right coronary arteries, there were 9 multi-vessel lesions. Detailed patient baseline characteristics are summarized in Table 1.

**Table 1:** Patient baseline characteristics

Parameter	Number of patients (54)
Age (y)	65 ± 8
Male sex	63% (34)
Weight (kg)	66 ± 11
Height (cm)	165 ± 8
Body mass index (kg/m^2^)	24 ± 3
Cardiovascular risk factors	
Systolic blood pressure (mm Hg)	131 ± 20
Diastolic blood pressure (mm Hg)	76 ± 14
Angina pectoris	20% (11)
Diabetes	19% (10)
Hypertension	57% (31)
Hyperlipidemia	7% (4)
Coronary CT angiography	
Agatston score, % (*n*)	
0–399	69% (37)
400–799	22% (12)
> 799	9% (5)
CCTA stenosis ≥ 50% (patient)	56% (30)
CCTA stenosis ≥ 50% (vessel)	65% (51)
AccuFFR ≤ 0.80 (patient)	37% (20)
AccuFFR ≤ 0.80 (vessel)	32% (25)
Vessel location, % (*n*)	
LM/LAD	58% (45)
LCX	13% (10)
RCA	18% (14)
Multi-vessels	11% (9)
Stenosis degree, % (*n*)	
< 50%	43% (23)
≥ 50%	57% (31)
FFR ≤ 0.8	41% (22)

*CT* computed tomography, *CCTA* coronary computed tomography angiography, *LM* left main artery, *LAD* left anterior descending artery, *LCX* left circumflex artery, *RCA* right coronary artery, *FFR* fractional flow reserve

### Correlation and agreement between AccuFFRct and invasive FFR

**Fig. 1:**
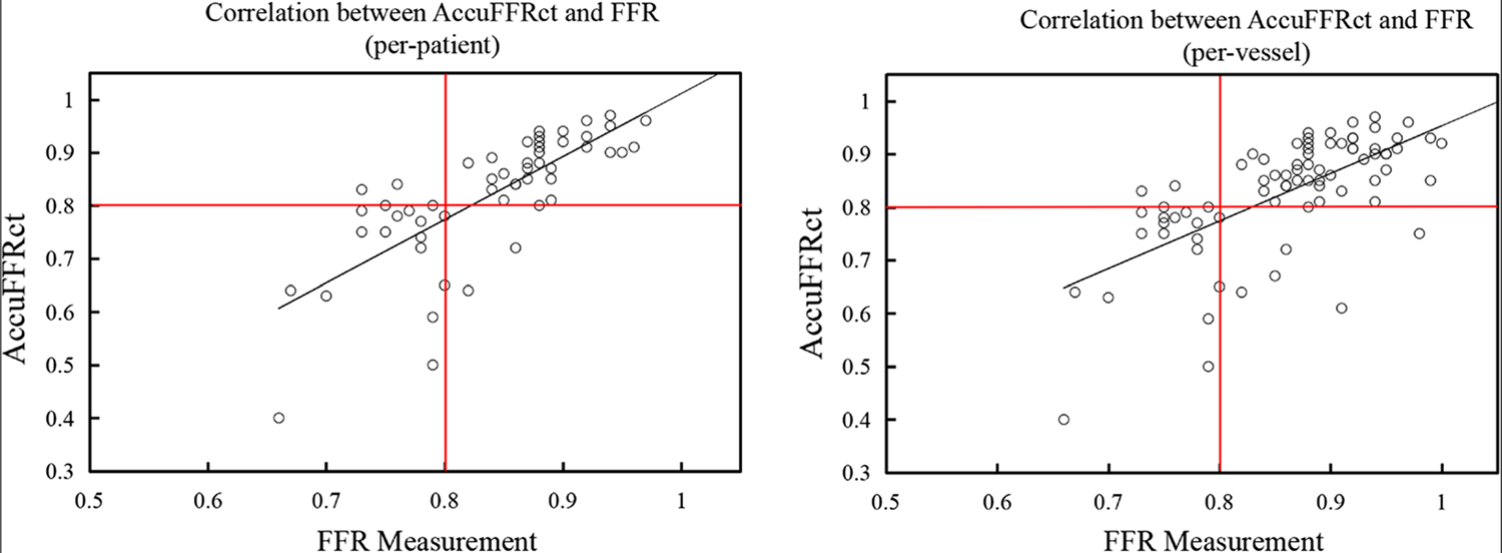
Per-patient correlations (*r* = 0.76, *p* < 0.0001) and per-vessel correlations (*r* = 0.65, *p* < 0.0001) between wire-based FFR and AccuFFRct. *FFR* fractional flow reserve

**Fig. 2:**
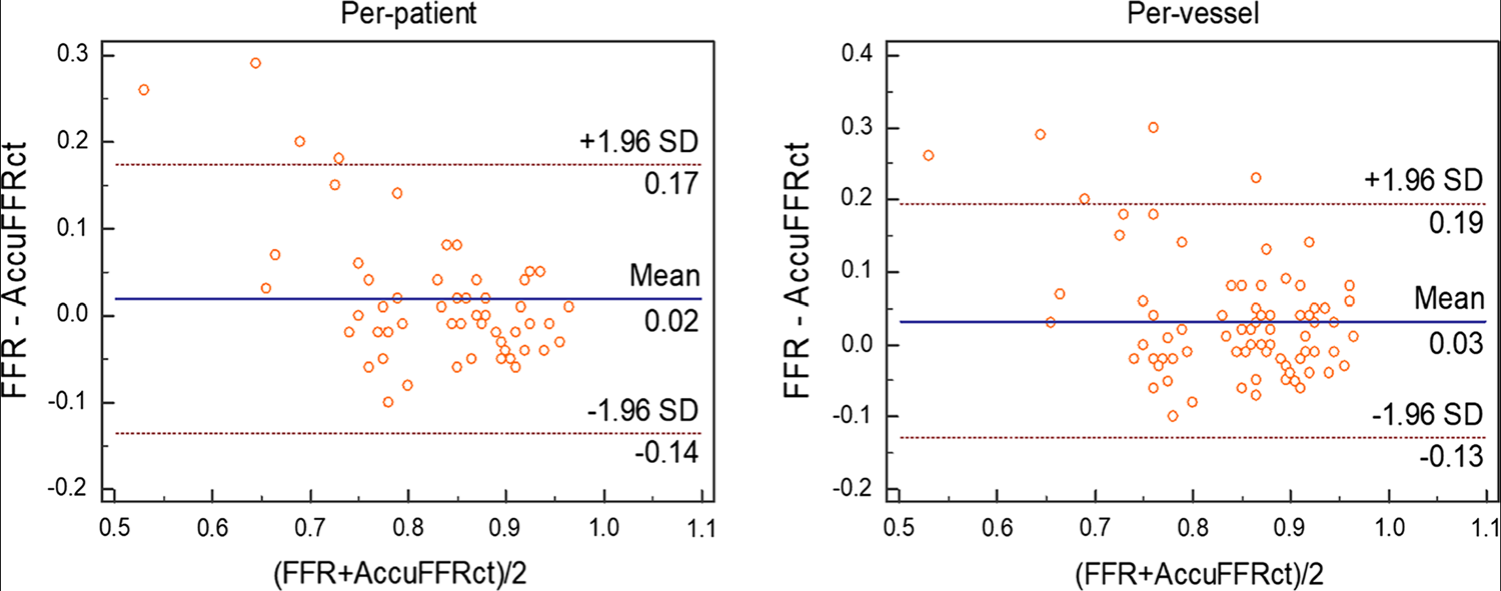
Bland–Altman plot of FFR and AccuFFRct on the per-patient and per-vessel basis, respectively. FFR = fractional flow reserve

**Fig. 3:**
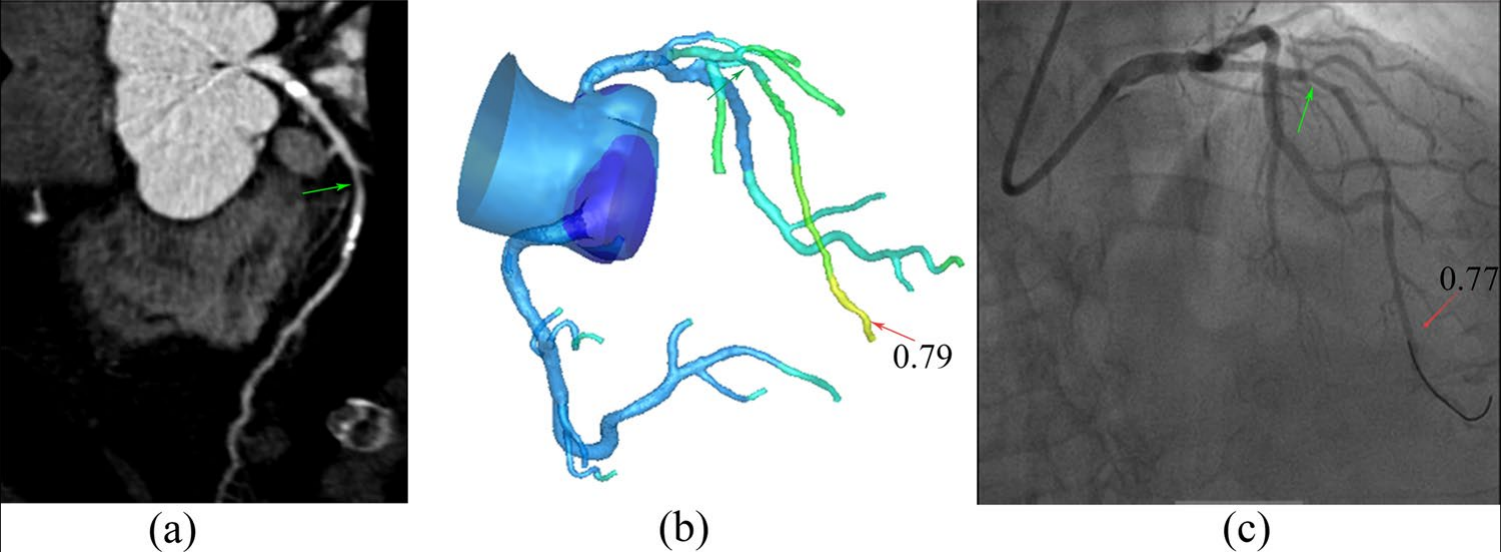
AccuFFRct results with invasive FFR measurement. **a** CCTA demonstrating 80% stenosis at the proximal to middle portion of LAD (green arrow); **b** a computed AccuFFRct value of 0.79 (red arrow); **c** the corresponding measured FFR value of 0.75, demonstrating stenosis ischemia. *FFR* fractional flow reserve; *CCTA* coronary computed tomography angiography, *LAD* left anterior descending artery

The correlation and agreement between AccuFFRct and FFR on a per-patient and per-vessel basis are shown in Figs. 1 and  2, respectively. Good correlation (Pearson’s correlation coefficient *r* = 0.76 and 0.65, *p* < 0.0001) and agreement (mean difference 0.06 ± 0.07) were observed. Representative patient case from the study is shown in Fig. 3.

The average AccuFFRct was 0.83 ± 0.10. Among all 78 vessels, there were 20 true positives (TP), 50 true negatives (TN), 6 false positives (FP), and 2 false negatives (FN) using FFR as the reference standard. With AccuFFRct ≤ 0.8 taken as the cut-off value of ischemia-causing stenoses (FFR ≤ 0.8), the sensitivity, specificity, positive predictive value (PPV) and negative predictive value (NPV) of AccuFFRct for diagnosis of ischemia-causing stenoses on the per-patient basis were 89.5, 91.4, 85.0, and 94.1%, respectively. The diagnostic accuracy was 90.7%. For the per-vessel assessment, the diagnostic accuracy, sensitivity, specificity, PPV, and NPV for AccuFFRct were 89.7, 90.5, 89.5, 76.0 and 96.2%, respectively. Detailed information of diagnostic performance of AccuFFRct is summarized in Table 2. When using CCTA only to predict FFR ≤ 0.8, the accuracy, sensitivity, specificity were 38.9, 100.0, 5.7% on per-patient basis and 32.0, 100, 7.0% on per-vessel basis, respectively. It is notable that AccuFFRct showed much better diagnostic performance in detecting ischemia-causing stenoses than the traditional assessment by CCTA. The extremely low specificity and accuracy of CCTA means overestimation of lesion severity, which may lead to unnecessary downstream invasive tests or intervention therapies. The high accuracy, sensitivity, specificity of AccuFFRct means it can get accurate assessment of coronary stenoses in most of cases without obvious underestimation or overestimation.

**Table 2:** Diagnostic performance of AccuFFRct for the prediction of ischemia of lesion on a per-patient and per-vessel level

Parameter	AccuFFRct ≤ 0.80 [95% CI]	AccuFFRct ≤ 0.80 [95% CI]
Per-patient level (*n* = 54)		Per-vessel level (*n* = 78)
Accuracy	90.7 [79.7–96.9]	89.7 [80.8–95.5]
Sensitivity	89.5 [66.9–98.7]	90.5 [69.6–98.8]
Specificity	91.4 [76.9–98.2]	89.5 [78.5–96.0]
PPV	85 [65.5–94.4]	76 [59.5–87.2]
NPV	94.1 [81.1–98.3]	96.2 [87.20–98.9]

Data are shown in percentage with raw data in parentheses and 95% confidence interval in brackets. *CI* confidence interval, *NPV* negative predictive value, *PPV* positive predictive value

**Fig. 4:**
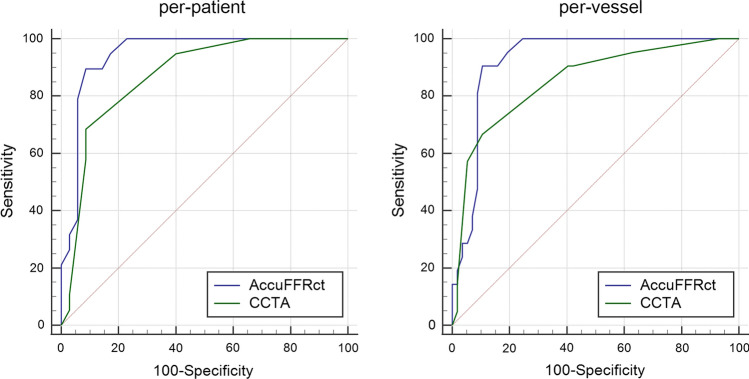
Areas under the curve (AUC) for receiver-operating characteristics (ROC) of AccuFFRct and CCTA, for per-patient and per-vessel basis. *CCTA* coronary computed tomography angiography

The ROC curves of AccuFFRct and CCTA on a per-patient and per-vessel basis are shown in Fig. 4. For a per-patient basis, the AUC for AccuFFRct and CCTA was 0.945 vs. 0.870, while for a per-vessel basis, the AUC for AccuFFRct and CCTA was 0.925 vs. 0.853. This also showed the superior diagnostic ability of AccuFFRct in identifying whether a stenosis can lead to ischemia.

## Discussion

We have developed a novel method that allows fast computation of FFR from CCTA images alone. In the study on a population of 54 patients with stenoses in 78 coronary vessels, AccuFFRct showed a good correlation with Pearson’s correlation coefficient *r* = 0.76 (*p* < 0.001) and agreement with invasive FFR. The overall diagnostic accuracy of AccuFFRct in predicting hemodynamically significant CAD (defined by FFR ≤ 0.80) was 90.7 and 89.7% on a per-patient and per-vessel basis, respectively. In this population, the accuracy of CCTA was only 61.8 and 58.1% for per-patient and per-vessel, respectively. The diagnostic accuracy of AccuFFRct is much higher than CCTA in the functional evaluation of lesion-specific ischemia. The cause is that CCTA often overestimates stenosis severity, and existing studies showed that less than 50% of CCTA-defined severe stenoses caused ischemia [2, 13]. Considering both anatomic and functional information, AccuFFRct can comprehensively assess the influence of stenoses on blood flow and facilitate clinical decision-making.

CT-derived FFR methods can estimate FFR from typically acquired CCTA images without additional image acquisition, CCTA protocols, and medication administration modification. The diagnostic performance of such approaches has been validated and verified [9–11, 14, 15]. A 3D CT-FFR technique (FFR_CT_, Heartflow) exhibited good correlations with invasive FFR, with diagnostic accuracy ranging from 73 to 86% and AUCs of FFR_CT_ in detecting lesion-specific ischemia ranging from 0.81 to 0.90 [9–11]. FFR_CT_ showed excellent diagnostic performance compared with CCTA, SPECT and PET and reduced more than one-half ICA in clinical practice [16, 17]. Hlatky et al. [18] demonstrated that the use of FFR_CT_ to guide ICA planning and revascularization could reduce costs and improve clinical outcomes. Furthermore, a recent trial including 5083 patients represented that FFR_CT_ could provide much more useful information for clinical decision-making and FFR_CT_-guided patient management enhanced one-year survival free of major adverse cardiac events [19, 20]. Besides, Siemens Healthcare [15] reported a new approach for computing cFFR from CCTA images using a reduced-order CFD model. The correlation of cFFR and invasive FFR ranged from 59 to 75% and the AUCs varied from 0.83 to 0.92 [15, 21, 22]. The machine learning (ML)-based cFFR was the latest version of the CT-FFR approach developed by Siemens Healthcare [23]. This algorithm learned the output of the CFD model and CCTA anatomies. Validation studies [12, 24] showed that the highest diagnostic accuracy of ML-based cFFR was 85%, with the sensitivity, specificity, PPV and NPV of 89, 76, 89 and 77%, respectively. The highest AUC of ML-based cFFR was 0.84. A more recent CT-FFR study (μCT-FFR) using transluminal attenuation gradient (TAG) to define boundary conditions also showed good results, with the diagnostic accuracy of 91% with sensitivity of 89% and specificity of 91% [25]. Besides CFD-based approaches, Gao et. al. proposed a deep neural network solution (TreeVes-Net) that allows obtaining FFR values directly from static coronary CT angiography images, with AUC = 0.92 for detecting FFR ≤ 0.8 [26]. In addition, blood was generally modelled as Newtonian fluid in most validated approaches, which was reliable because there was no essential difference in results between Newtonian and non-Newtonian fluid assumptions in large arteries [27, 28]. On the other hand, there were studies showing differences between non-Newtonian and Newtonian fluid flows [29, 30], and Toshiba proposed a CT-FFR technique with non-Newtonian fluid model [31], however, the diagnostic performance was not very good (accuracy of 83.9% with AUC of 0.88).

Our study demonstrated credible results using AccuFFRct for exploring ischemia-causing stenosis comparing with above-mentioned works. The overall diagnostic accuracy of AccuFFRct was 89.7% on the per-vessel basis, which is higher than that of FFR_CT_ and cFFR. The sensitivity, specificity, PPV, and NPV were 90.5, 89.5, 76.0 and 96.2%, respectively, while those for FFR_CT_ were 84%, 86%, 61%, and 95%, respectively. μCT-FFR showed good diagnostic performance with 1% higher accuracy, While AccuFFRct resulted in slightly better sensitivity. It should be noted that higher sensitivity means less false negatives, which were critical in clinical application because false-negative results might put patients in danger for wasting the best treatment time. AccuFFRct showed an AUC of 0.945 for a per-patient basis and 0.925 per-vessel basis for categorizing functionally significant stenoses. The AUCs for FFR_CT_, cFFR, ML-based cFFR, μCT-FFR and TreeVes-Net-based FFR were 0.9, 0.92, 0.84, 0.92, 0.92, respectively. One cannot say AccuFFRct was superior in defining ischemia, but AccuFFRct did show very competitive results among similar works, and the diagnostic performance could be further optimized with future studies focusing on computational condition settings and algorithms, etc.

Except for the good diagnostic performance of AccuFFRct, the advantage of AccuFFRct lies in its time-efficient and convenient workflow. The application of FFR_CT_ requires significant computational time (1–4 h) due to the need of data transfer to off-site supercomputers [11]. Workstation-based cFFR takes approximately 40 min with ML-based CT-FFR and 43 min with the reduced-order CFD-based CT-FFR [12, 15, 24, 32, 33]. As for AccuFFRct, only 35 min is required for the whole workflow, including 3D reconstruction of coronary artery geometry and CFD simulation, on average. Time efficiency is a critical factor for patients with coronary stenosis during the diagnostic procedure, because the time allowed for them varies from several minutes to weeks depending on their symptoms and the severity of ischemia, so it is important to shorten the diagnostic time for helping more patients during a fixed time. Moreover, a new ML-based algorithm for 3D reconstruction is under development, the total duration of AccuFFRct calculation will be reduced to about 10 min in the coming new version.

Though invasive FFR measurement is relatively safe in clinical use, risks and other limitations are still inevitable. Kumsars et al. [34] reported that about 1/10 of FFR could not be obtained due to side branches or wiring failure. Moreover, the high price of pressure wires may become a financial burden for patients. As a result, advanced computational analytical approaches that can compute FFR values from CCTA images alone could lead to widespread clinical utilization. AccuFFRct offers a time-efficient and accurate tool for FFR computation, and the visualized anatomic geometry of coronary tree can be used for subsequent clinical planning.

The main limitation of this study is its relatively small sample size with all CCTA data from a single center. A multi-centered study with large sample size is necessary to validate AccuFFRct further. In this study, the workflow and analysis were all performed by professionals. Thus training of normal medical staff is essential to ensure reliable execution of AccuFFRct analysis.

## Conclusions

The novel CFD-based CT-FFR method, AccuFFRct, is influential in determining significant functional CAD in a time-efficient manner with good diagnostic performance. It may emerge as a safe, efficient, and credible tool for evaluating coronary stenosis severity during diagnostic CCTA. In the future, algorithms would be continuously updated, some computational conditions or assumptions could also be optimized to assure clinically reliable results and to help more patients. A multicenter prospective clinical trial would be conducted to study the accuracy and clinical suitability of the algorithm for different types of stenosis, such as left main lesion, tandem lesion and bifurcation lesion.

## Methods

### Study design

This was a retrospective, observational, analytical study conducted at The Second Affiliated Hospital of Zhejiang University School of Medicine. Patients who had undergone coronary computed tomography angiography (CCTA) and invasive FFR measurements within 2 months were included. This study was approved by the Institutional Review Board of the hospital and informed consent of the patients was waived.

### Study population

The study population comprised 54 stable patients with suspected or known CAD who underwent CCTA and FFR measurements between January 2016 and September 2017. Exclusion criteria included individuals with prior myocardial infarction, prior coronary artery bypass surgery (CABG), prior stenting at the lesion of interest, significant motion or blurring artifact in CCTA, occlusion in any major coronary artery.

### CCTA acquisition and analysis

**Fig. 5:**
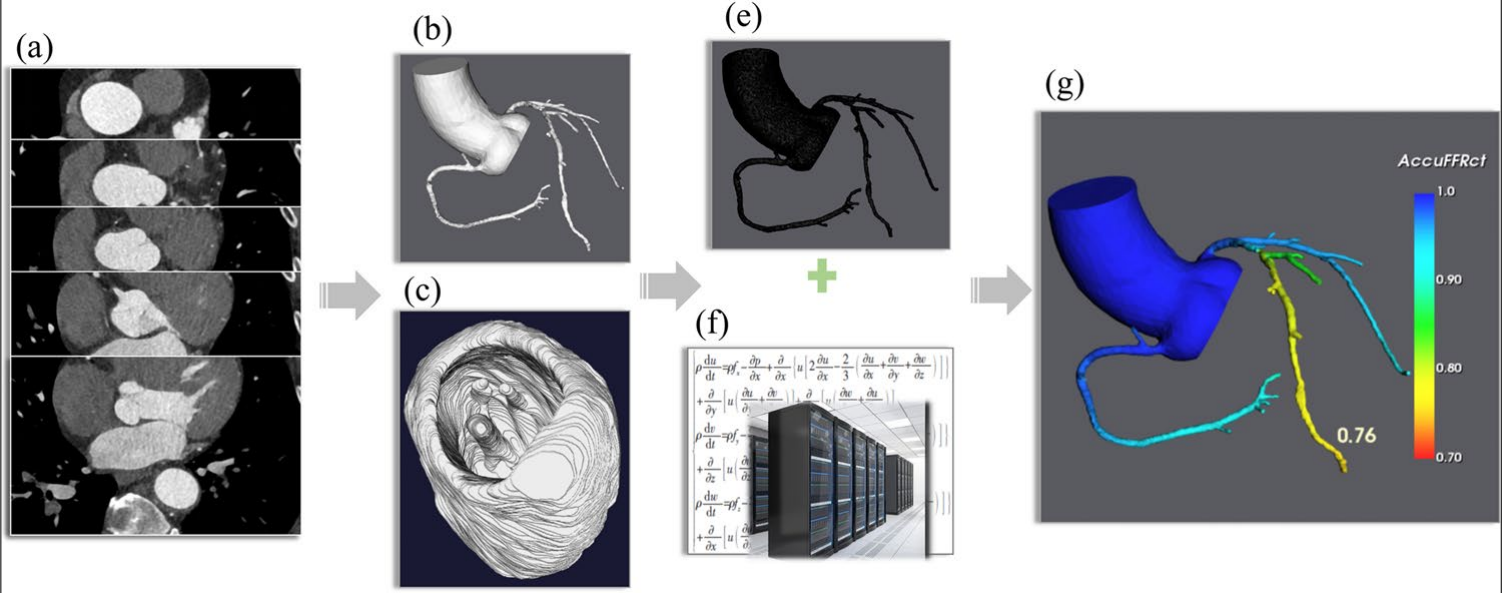
Flowchart for computing AccuFFRct: **a** CCTA image data; **b** segmented 3D coronary artery model; **c** segmented 3D ventricle model; **d** mesh generation; **e** coronary flow computational algorithm for computing coronary flow, pressure from Navier–Stokes flow governing equations; **f** AccuFFRct distribution over the coronary artery tree. **g** *CCTA* coronary computed tomography angiography, *3D* three-dimensional

CCTA was performed using a dual-source 128-slice CT scanner (Somatom Definition FLASH, Siemens, Forchheim, Germany). Medications were administered individually and different CCTA scan modes were chosen according to patients’ heart rate conditions. The tube voltage and tube current were set based on the body mass index of the patients. All CT images were reconstructed with a slice thickness of 0.5 mm. The CCTA image data was assessed in the central AccuFFRct core laboratory (ArteryFlow Technology Co., Ltd., Hangzhou, China) and selected for subsequent AccuFFRct calculation and analysis. The flowchart of this study is displayed in Fig. 5.

### ICA and FFR measurement

Invasive coronary angiography (ICA) was performed within 30 days after the CCTA acquisition according to standard practice. FFR measurements were conducted after inducing the maximum hyperemia by intravenous injection of adenosine at 180 μg/kg/min. FFR was defined by dividing the mean pressure distal to the lesion by the mean aortic pressure under hyperemic condition, and an FFR value of ≤ 0.80 was considered hemodynamically significant. FFR data were transferred to the central AccuFFRct core laboratory for the following analysis by independent, blinded readers.

### CFD-based AccuFFRct computation

Using the most recent AccuFFRct analysis software (AccuFFR®ct, version 1.0, ArteryFlow), analysis was performed in a blinded fashion in the central AccuFFRct core laboratory.

The calculation of AccuFFRct includes four steps:

1. **Anatomical model reconstruction and segmentation**
  In this step, the anatomical geometry model of coronary arteries and ventricles can be obtained accurately from CCTA image data. First of all, the fast marching algorithm and colliding fronts algorithm were applied to segment aorta and coronary tree, differentiating them from other anatomic parts which were also include in CCTA image data. Subsequently, the optimal vessel borders were identified using the level-set method base on segmented CCTA images to ensure the real vessel shape. Then with an automatic geometry cleaning algorithm, the anatomical model of the coronary tree was obtained by marching cubes method [35] and the left ventricle model was extracted by a deep-learning segmentation method based on an 8-layer residual U-Net to compute the myocardium volume further. As shown in Fig. 5a–c.
  In addition, though the CT images included in this study were relatively good, general CT data that meet the standard of Digital Imaging and Communications in Medicine (DICOM) with a slice thickness < 1 mm is compatible with AccuFFRct, which means our technique can be used in numerous hospitals.
2. **Mesh generation and boundary conditions**
  The anatomical model of coronary arteries should be pre-processed, including hole checking, smoothing, boundary face editing of the 3D model, and then it would be transformed into a mesh model for later numerical CFD simulation to obtain the flow field of blood. In general, millions of mesh elements with different sizes were generated based on the structure of an anatomical model. In general, the maximum mesh size was limited as 0.3 mm for the best balance of computational precision and time based on previous mesh sensitivity tests, coarser mesh may lead to bigger error. Boundary conditions were the physical and physiological conditions applied to the mesh model to simulate the interaction between coronary arteries and related body parts. In this study, the patient-specific blood flow and aortic pressure were set as the inlet boundary condition. The resting blood flow was estimated by myocardium mass (*Q* ∝ *M*^*k*^) and the flow rate was 2–4 times of resting condition at hyperemia. The blood flow rate at the outlet of each branch was calculated using Murray’s law (*Q*_*i*_ ∝ *d*^3^), and the rigid wall with no-slip boundary condition was applied to the vessel wall.
3. **CFD simulation**
  The flow field information, including pressure and velocity at each mesh element, could be calculated by solving the fluid governing Navier–Stokes equations using the finite volume method. In this study, blood was modeled as Newtonian fluid (density *ρ* = 1056 kg/m^3^, viscosity μ = 0.0035 Pa·s) and incompressible [27], a modified solver based on an open-source CFD package (OpenFOAM 7) was used for this simulation of coronary blood flow.
4. **AccuFFRct computation**
  The AccuFFRct value was calculated as the ratio of the distal pressure located at the measuring point of FFR to the mean aortic pressure. The simulation time for each case was approximately 35 min on a standard desktop, including the time of 3D reconstruction and CFD computation using AccuFFRct software.

### Statistical analysis

Continuous variables were presented as means ± standard deviations or median in case of non-normal distribution. Categorical variables were presented as frequencies and percentages. The diagnostic performance of AccuFFRct (the ability of AccuFFRct to predict whether the FFR ≤ 0.80 or FFR > 0.80, i.e., whether the lesion would cause ischemia or not) was evaluated by calculating sensitivity, specificity, PPV, NPV and overall accuracy. Using FFR as a reference standard, sensitivity was defined by true positives divided by the sum of all true-positive and false-negative cases, specificity was defined as true negatives divided the sum of all true-negative and false-positive cases, PPV = TP/(TP + FP), NPV = TN/(TN + FN) and accuracy = (TP + TN)/(TP + FP + TN + FN). Pearson correlation was used to quantify the degree of correlation between invasive FFR and AccuFFRct in detecting ischemia-causing stenoses. The area under the curve (AUC) derived from receiver-operating characteristic (ROC) curve analysis were computed using invasive FFR as the reference standard for checking classification performance (positive or negative for ischemia-causing) of AccuFFRct. The Bland–Altman statistics were also performed to assess the agreement of FFR and AccuFFRct. A two-sided P value of less than 0.05 was considered statistically significant. All statistical analyses were performed using MedCalc (MedCalc Software Inc., Belgium).

## Data Availability

The datasets used and analyzed during the current study are available from the corresponding author on reasonable request.

## Abbreviations

PCI: Percutaneous coronary intervention
CABG: Coronary artery bypass grafting
CCTA: Coronary computed tomography angiography
CAD: Coronary artery disease
3D: Three-dimensional
CT: Computed tomography
CFD: Computational fluid dynamics
ICA: Invasive coronary angiography
FFR: Fractional flow reserve
ML: Machine learning
PPV: Positive predictive value
NPV: Negative predictive value
AUC: Area under the curve
ROC: Receiver operating characteristic
